# Supplementary figures and images for: A conserved expression signature predicts growth rate and reveals cell & lineage-specific differences
Source: PLoS Comput Biol. 2021 Nov 11;17(11):e1009582. doi: 10.1371/journal.pcbi.1009582 (PMC8610284; doi:10.1371/journal.pcbi.1009582)

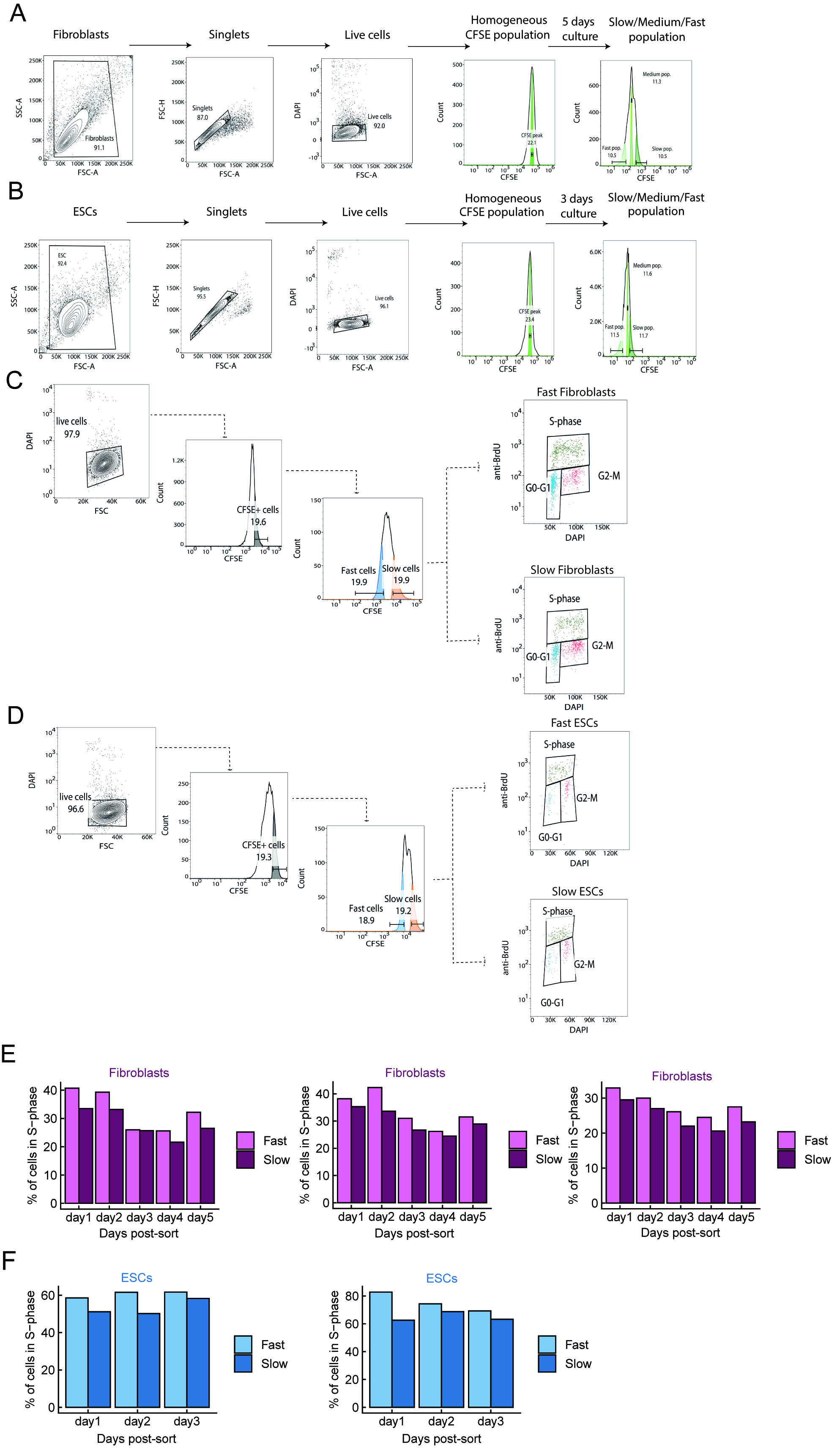

Supplement: S1 Fig — FACS gating strategy for CFSE staining to get cell subpopulations with different proliferation rates. (A, B) The gating strategy for CFSE staining to get cell subpopulations with different proliferation rates in fibroblasts (A) and in ESCs (B). Slow, medium and fast proliferating cell subpopulations were sorted by FACS according to their CFSE signal. Then RNA-seq was performed on each of the three subpopulations. (C, D) FACS gating strategy for measuring the heritability of proliferation rates. The gating strategy for measuring the heritability of proliferation rates in fibroblasts (C) and in ESCs (D). In all experiments, the laser voltage was increased so that, when sorting high and low CFSE cells, the modal CFSE signal was at least 103; the voltage is not the same for the first and second CFSE sorts. (E) 3 Replicates of fibroblasts that similar to Fig 1E. (F) 2 Replicates of ESCs that similar to Fig 1F. (TIF) [file pcbi.1009582.s001.tif]

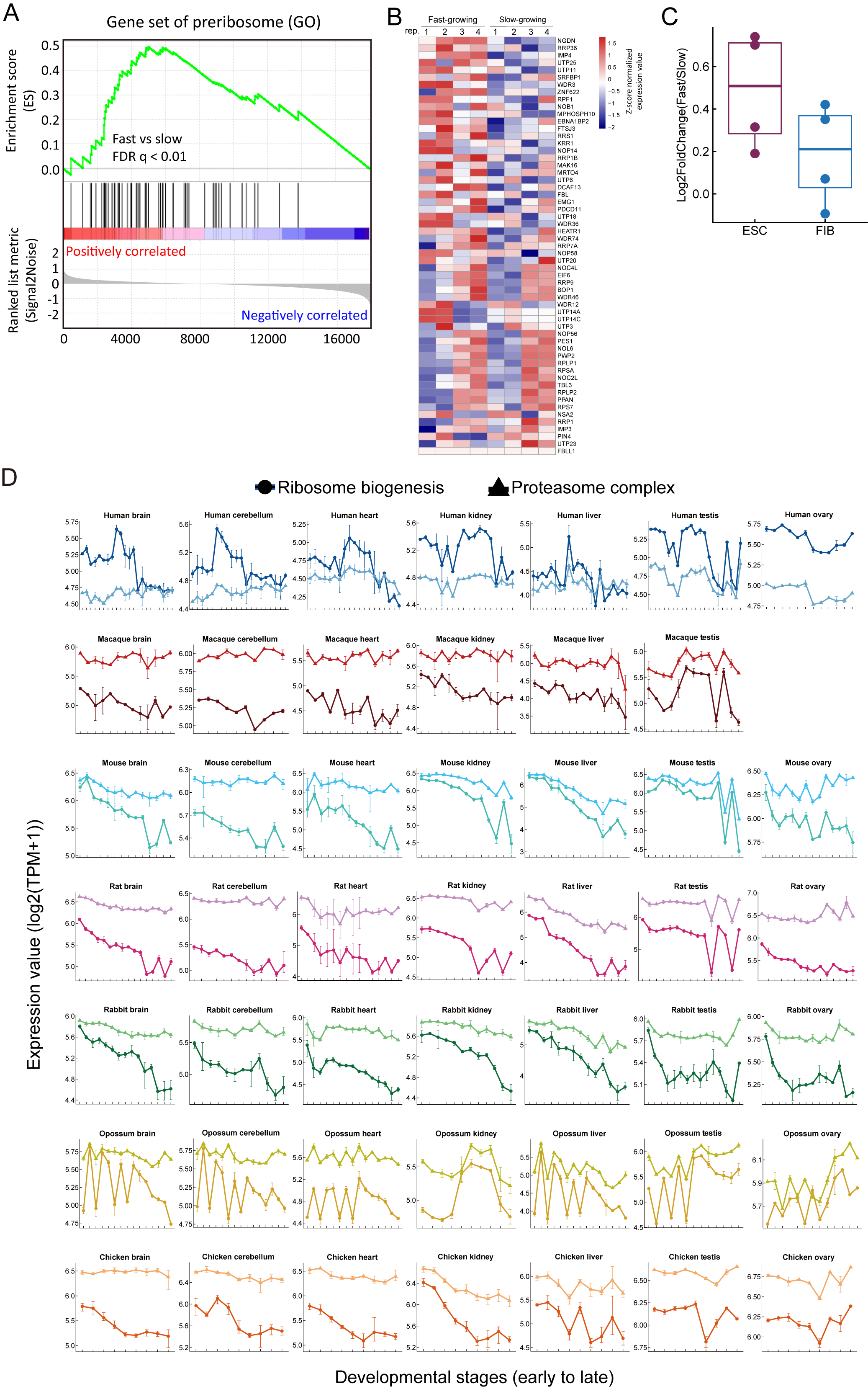

Supplement: S2 Fig — (A) GSEA result plot of Go preribosome genes set for ESC. (B) The heatmap shows the expression (z-scored read counts) of preribosome genes in ESCs across four biological replicates of the CFSE sorting experiment. (C) Higher expression of Myc in both fast proliferating ESCs and fibroblasts. log2 fold change of Myc expression between fast and slow proliferating subpopulation in both ESCs and fibroblasts, each cell type 4 replicates. (D) Correlated changes in the expression of ribosome biogenesis and proteasome related genes during organ development. Change of average expression of log2(TPM+1) of genes in ribosome biogenesis (Go preribosome) gene set and proteasome complex (Go proteasome complex) gene set with developmental stages across different organs in seven species [16]. Points (circle and triangle) are the mean expression of replicates, error bars represent the maximum and minimum value in the replicates. (TIF) [file pcbi.1009582.s002.tif]

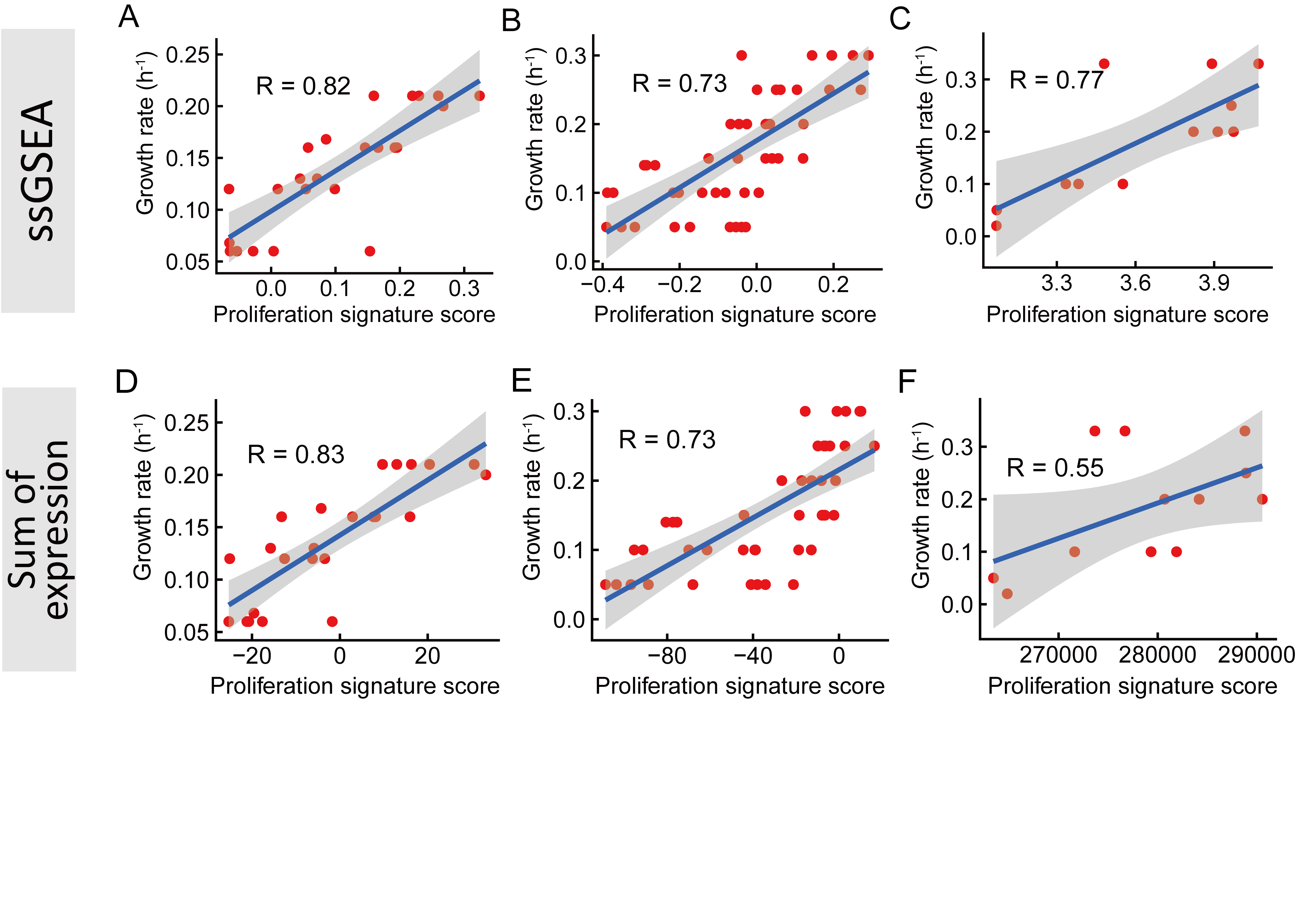

Supplement: S3 Fig — (A-C) Using the Normalized Enrichment Score from ssGSEA to predict growth rate in three different data sets. The Pearson correlation of proliferation signature score with growth rate in are R = 0.82 (p = 8.9×10^−7^), R = 0.73 (p = 1.3×10^−8^) and R = 0.77 (p = 3.7×10^−3^). (D-F) Similar to A-C, but using the sum of expression values for all genes in the proliferation signature gene set to calculate proliferation signature score. The Pearson correlation of proliferation signature score with growth rate are R = 0.83 (p = 7.0×10^−7^), R = 0.73 (p = 1.6×10^−8^) and R = 0.55 (p = 0.65×10^−2^). (TIF) [file pcbi.1009582.s003.tif]

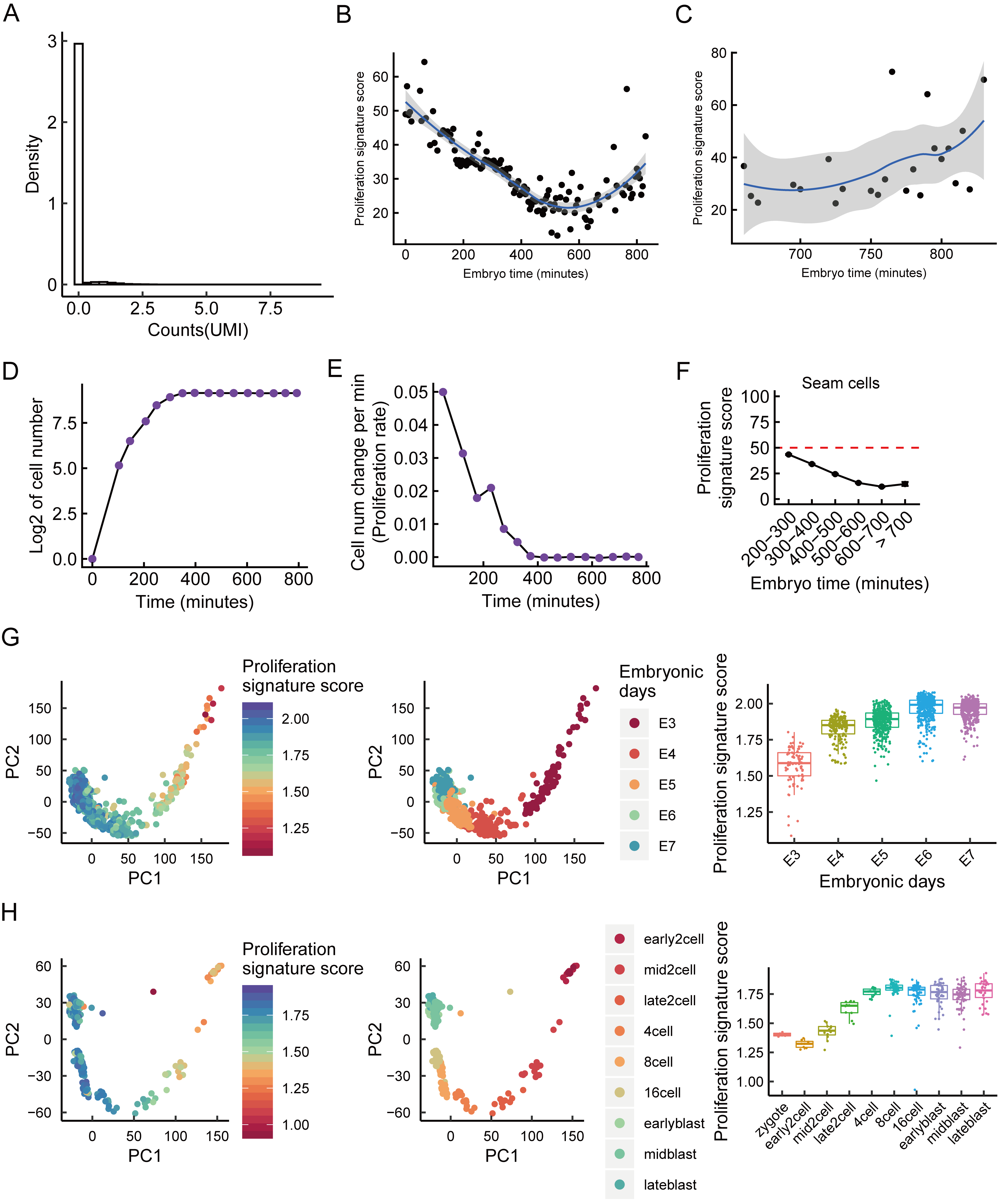

Supplement: S4 Fig — (A) A density histogram of counts (UMI) across 1000 randomly sampled cells; 95.6% of genes have zero reads. This causes ssGSEA to give unreliable results, so the sum of expression values method is used for calculating the proliferation signature score for single cells. (B) Cells are binned by embryo time, and the mean proliferation signature score for all cells not the three outlier cell types (germline, intestine and M cells). The Spearman correlation is rho = -0.73 (p = 8.7×10^−24^) for binned data, and rho = -0.45 (p < 2.2×10^−16^) for unbinned data. (C) Similar to Fig 5C, but only showing cells with an age higher than 650 minutes, rho = 0.5 (p = 1.5×10^−2^). (D, E) The change in cell number, and the rate of change in cell number, during development, as measured by microscopy [70]. (F) Change in proliferation signature score for seam cells, which form multinucleated cells through cell-fusion. (G) Human single-cell gene expression data from Petropoulos et al. [72] projected onto the first two principal components and colored by proliferation signature score or developmental stages. And boxplot shows the change of proliferation signature score with developmental stages. (H) Similar to G, but using mouse scRNA-seq data from Deng et al. [73]. (TIF) [file pcbi.1009582.s004.tif]

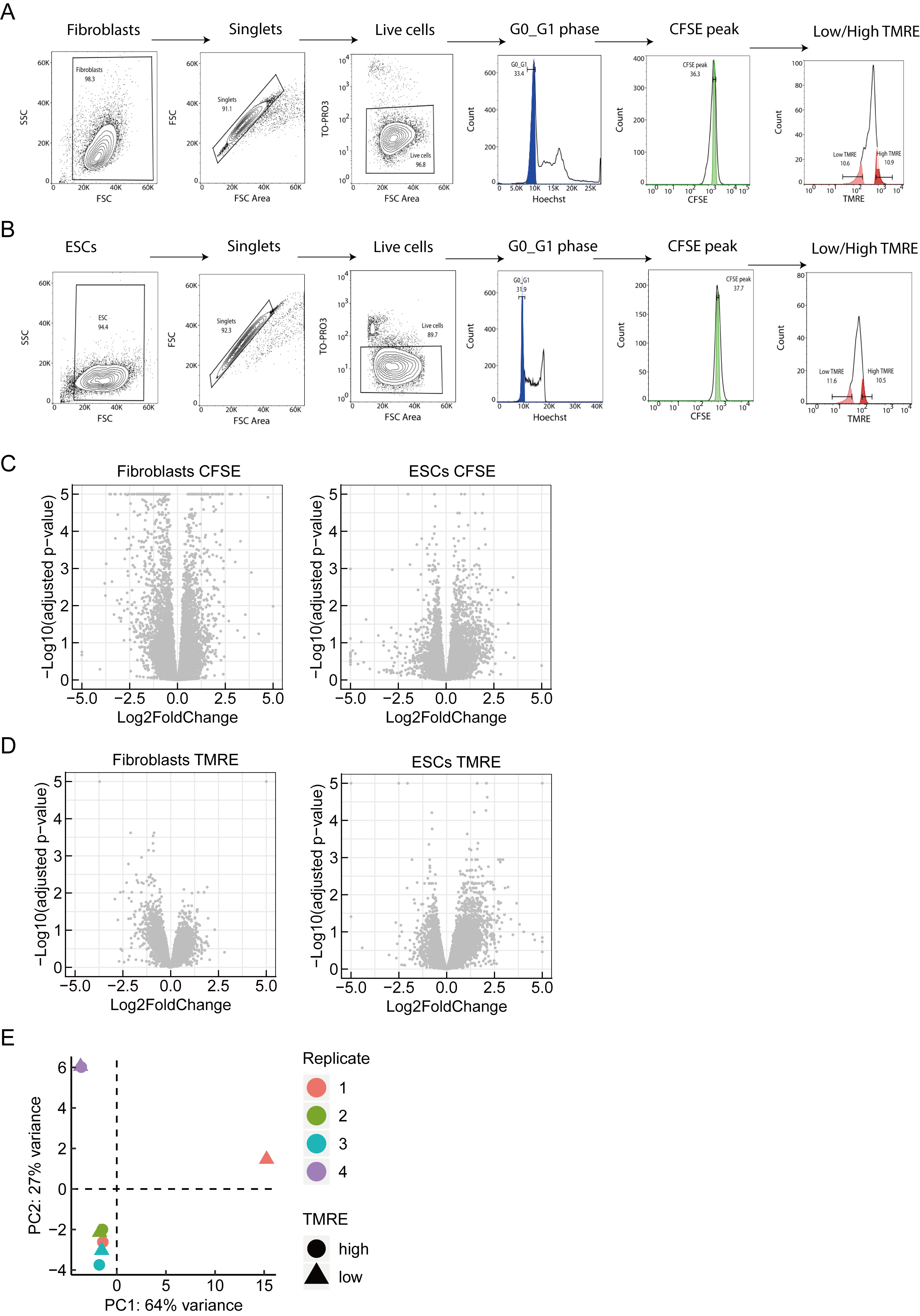

Supplement: S5 Fig — (A, B) The gating strategy for TMRE staining to get cell subpopulations with different mitochondrial states in fibroblasts (A) and in ESCs (B). We use Hoechst to get cells in G0/G1, gate by CFSE to get a more uniform cell population, and separate populations with high and low TMRE signal, then do RNA-seq on each of the two subpopulations. (C, D) Deseq2 was used to calculate log2 fold change and adjusted p-values for CFSE sorting (C) and TMRE sorting (D), combining biological replicates. To set the axes to be maximally informative, genes with p < 10−5 had p set to 10−5, and those abs(log2 fold change) > 5 were truncated at -5 or +5. (E) PCA for RNA-seq data of ESCs sorted by TMRE. Low TMRE ESCs of replicate 1 is an outlier, so we remove replicate 1 for all analysis. (TIF) [file pcbi.1009582.s005.tif]

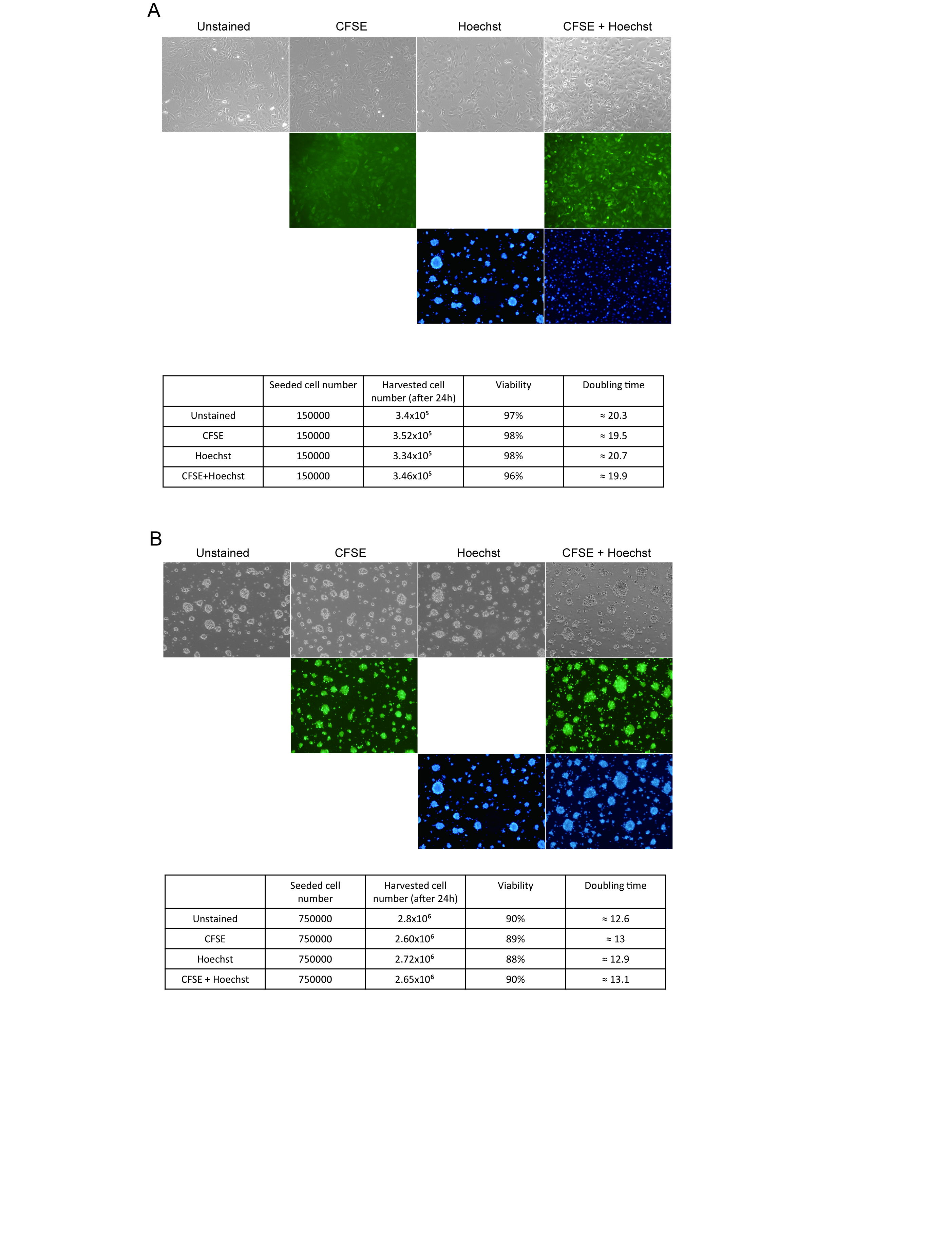

Supplement: S7 Fig — Shown are the estimated doubling times (based on the increased in cell number after 24hrs growth) and measured viability (trypan blue) for fibroblasts (A) and ESCs (B). The microscopy shows that stained cells maintain the correct morphology. (TIF) [file pcbi.1009582.s007.tif]

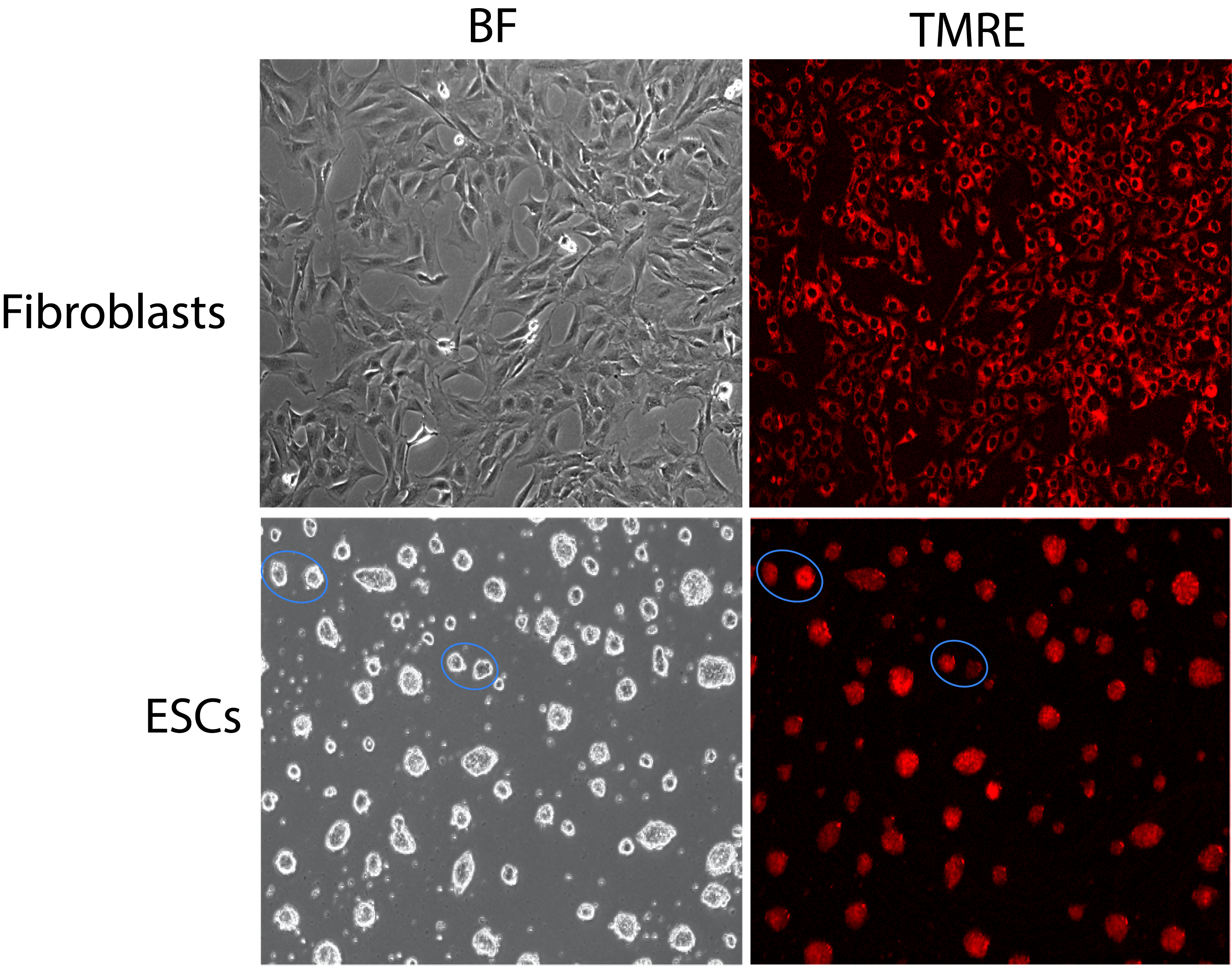

Supplement: S9 Fig — Bright-field and TMRE staining images for both Fibroblasts and ESCs. Two pairs of ESC colonies of similar size but showing staining heterogeneity are circled in blue. (TIF) [file pcbi.1009582.s009.tif]
