# Supplementary material for: A conserved expression signature predicts growth rate and reveals cell & lineage-specific differences
Source: PLoS Comput Biol. 2021 Nov 11;17(11):e1009582. doi: 10.1371/journal.pcbi.1009582 (PMC8610284; doi:10.1371/journal.pcbi.1009582)
Supplement: Attachment — Submitted filename: Response to Reviewers - PLOS Comp Bio - 2021 0627 f+BP.docx [file pcbi.1009582.s021.docx]

**Reviewer #1**: Jiang et al present a clever experimental approach to measuring growth rate-dependent gene expression, and find a gene-expression profile that predicts growth rate across a range of species and cell types. The experimental approach is to label cells with a stable, cell-permeable dye, sort the cells to get a homogeneously fluorescent starting population, and then sort cells at a later time for RNA-seq according to how much fluorescence remains because fluorescence decreases as cells grow and divide). Overall, the experiments are rigorously performed and the predictive power is impressive, especially with cross-species validation. The paper will be an important contribution to the study of cell-to-cell heterogeneity and growth control. However, I do have two main recommendations to strengthen it:

We thank the reviewer for the positive feedback and the constructive recommendations on how to improve the manuscript.

1) The manuscript could do a better job of placing this work in the context of the existing literature. The work of Terry Hwa and colleagues on the connection between translational capacity and growth is not cited. Recent work identifying semi-heritable cell-to-cell heterogeneity in mammalian cells using RNA-seq is not cited (Raj lab and Tanay lab). Wytock & Motter 2019 is cited, but other work on predicting growth rate from gene expression is not (e.g., doi:10.1371/journal.pone.0075320 which discusses ribosomal protein expression).

We thank the reviewer for pointing out those omissions. We have now mentioned and cited the papers from the Hwa, Raj and Drummond labs as suggested.

2) The computational/prediction method should be more completely discussed in the main text and the method should be more explicitly justified. For example, the cutoff for growth-correlated genes of rho = 1 seems a bit confusing at first (line 255), until one considers that this is a rank (Spearman) correlation and there are only three bins of growth rate (cells sorted with high, medium or low fluorescence). So rho = 1 just means growth rate increases monotonically with growth. This seems sensible enough, but it does not consider measurement noise the way a proper statistical model would, so this decision should be defended. (And because of experimental noise, it also would likely cause problems if, say, another experiment used 5 bins because the cutoff would likely be too stringent.) The addition of ribosome-biogenesis and proteasome gene sets to the genes that contribute to the proliferation signature score is also somewhat arbitrary (or at least one could imagine different decisions being made). So some kind of analysis of how the proliferation score is sensitive (or not) to these decisions is warranted. The performance of the proliferation score is well validated by using data from divergent organisms, but that's not to say it could not be improved. Likewise, how the gene-expression signature is transferred from one organism to another is not fully described. The Methods section explains that homologs were identified using the R package Biomart, but more detail is needed. Are these 1:1 orthologs? orthologs + paralogs? It is also not entirely clear how the C. elegans profileration predictions were tested against data. It seems that the comparison is only based on the rate of increase of cell number through development. Are there C. elegans data on the actual rates of division of particular cells that could be used as well?

1) Yes, we do think our cut-off is strict enough, as we only use genes increasing monotonically with growth in both two fib replications and two ESC replications.

If there are more bins, say 5 bins, then rho can be set smaller. But as we only have 3 bins and set threshold as rho=1 gave us 243 genes, which is a lot of genes. Using rho=1 also removes any arbitrary thresholds (for p-values or for rho itself).

2) We used ribosome-biogenesis gene sets and proteasome-related gene sets because we first found them to be enriched in fast proliferating cells among fibroblasts, ESC and yeast. None of the gene sets were enriched in slow proliferating cells for all three: fibroblasts, ESCs and yeast **(Figure 3C, D)**.

3) For the mapping of genes across different species, we used the R package Biomart. If the mapping from human to other species is one to many, then we chose the first mapped gene. If the mapping is many to one, then we kept the gene replication.

4) “Are there *C. elegans* data on the actual rates of division of particular cells that could be used as well?”. To our knowledge, there is no such kind of data available yet.

**Reviewer #2**: This paper used a fluorescence-activated cell sorting (FACS) based method to sort fast- and slow-proliferating subpopulations in mammalian cells and reports a gene expression signature, which can be used to predict the proliferation rates across eukaryotic species and cell types. This signature includes expression of house-keeping genes such as ribosome and proteasome, which are often expressed at high levels and hence can be reliably measured even using methods with low sampling efficiency (such as single-cell RNA-seq). This proliferation model is more robust than other models currently available and it has the potential to be widely used, particularly in the field of single-cell transcriptomics. Additionally, the authors found that slow-proliferating ESCs display a more naïve pluripotent expression profile than fast-proliferating ESCs. This finding is counter-intuitive and should be of interest to the stem cell field. Overall, the paper is well written, the data are well presented and supports the main collusion.

We thank the reviewer for the overall positive assessment of our manuscript.

The weaker part of the paper is the cell-type specific correlation between mitochondria function and proliferation rates, where the authors seem to have made efforts towards gaining mechanistic insights behind the correlation. But the data seem piecemeal and it is unclear what message these data really deliver, particularly with seemingly contradicting data unexplained (see specific comments). Compared to other important findings in the paper, this part could perhaps be reduced to a more coherent and concise figure.

We have followed the reviewer’s advice and have made changes to this part of the manuscript according to the specific comments 7-9 below.

Specific comments:
1. What effect does protein degradation have on the intensity of CFSE dye? The authors comment that protein degradation should not have a huge effect but it should be shown with analytical/experimental evidence, especially given the finding that proteasome genes are differentially expressed between the two sorted populations. This may be tested experimentally using proteasome inhibitors.

CFSE dye binds to intracellular lysine residues and thus may be affected by protein degradation (depending on if the dye signal is quenched or destroyed during protein degradation). However, our measurements of doubling time of fibroblasts and ESCs using CFSE signal gave a good result **(Figure 1C,D)**, suggesting the impairment of CFSE in fast proliferating cells by higher expression of proteasome is negligible when using CFSE as a signal of proliferation. For that reason, many studies use this method, as for example Methods (2015) 82:29-37 and Oncogene (2009) 28:4353-4363. Also, testing using proteasome inhibitors may not fully replicate the conditions in unperturbed exponentially growing cells (doi:10.1016/S0021-9258(19)63348-X).

2. Given that the entire paper is built on sorting using CFSE dye, an orthogonal method should be used to validate that the sorted slow proliferating cells are indeed proliferating more slowly. I envision that a simple growth curve should be sufficient.

We do show that the sorted slow proliferating cells remain slower for at least three days than the fast proliferating cells for both fibroblasts and ESCs **(Figure 1E,F and S1E,F)**. The measurement in these figure panels is % of cells in S-phase based on BrDU incorporation, which is an orthogonal method of proliferation speed measurement compared to the CSFE dye.

3. Line 729, why only the genes with “mean(Cor(gene(i), pr(i))) = 1” are chosen as “proliferation correlated genes”? The genes with correlation value of -1 should be also highly correlated and equally important as the value 1 genes.

No gene sets were enriched in slow proliferating cells for all three: fibroblasts, ESCs and yeast **(Figure 3C, D)**. This suggests that most negatively correlated genes are cell type specific, not stable, or of low magnitude difference between fast and slow cells, and thus might not be good candidates for robust proliferation prediction across cell types or species.

4. It would be useful to know which (groups of) genes in the selected gene sets contribute more for predicting the proliferation rate.

In a gene set with 100 genes, there are 10^29 subsets of size 50, and the same number for size 49, and 51. Because any allowed subgroups size (1 – 100) is allowed, there are an infinite number of possible groups, within just a single gene set. Testing at the sub-gene set level leads to a combinatorial explosion in the number of free parameters, and would require dozens, if not hundreds, of biological replicates in order to make statistically significantly sound conclusions.

This curse of dimensionality is the main reason that previous models are overfit, resulting in poor performance on new data (e.g., Wytock & Motter 2019). By limiting our analysis to pre-defined gene sets, we avoid this problem, resulting in a model that accurately predicts proliferation rates, instead of being overfit to the training data.

To properly draw conclusions from the data, it is essential to avoid this explosion of dimensionality, and thus we limit our analysis to predetermined externally defined gene sets.

5. Contradicting statement on the relation between % S-phase cells and proliferation rates. Line 178, higher S phase fraction is used to support that fast proliferating subpopulations maintain higher proliferation rates (Figure 1 E, F). This conclusion can be also found in Line 422-423. However, in Line 293-296, for explaining no correlation between PCNA or Ki67 expression with proliferating rate, authors mentioned that “these markers are measuring the fraction of the population that is in S-phase or is not in G0…”

We now deleted the statement “these markers are measuring the fraction of the population that is in S-phase or is not in G0…”. Growth rate difference can not be detected by the expression level of PCNA or Ki67, as the difference is intrinsic heterogeneous and too small to be reliable. That's why we need a set of genes to do the prediction but not only PCNA or Ki67.

6. Magnitude of the proliferation score. I noticed that the proliferation score varies from 10-1 level (yeast, Figure 4B) to 102 level (human, Figure 5G). I am also curious that sometimes this score become negative. What does this mean? Can the authors comment on the condition where this score might be compared?

The range of the values calculated depends on the source and normalization method of the source expression data. The range of data from microarray (yeast, Figure 4B) differ from Illumina RNA-seq (human, Figure 5G). Also, data normalized to RPKM/FPKM differ from expression counts data. While the data ranges and absolute values differ among source data, the method itself is robust to data from different sources and different normalization methods.

7. Antimycin and oligomycin are both mitochondria respiration inhibitors, yet the two have opposite effects on fast vs. slow fibroblasts (in Fig. 7C antimycin inhibits proliferation of slow FIB greater than fast FIB, but in Fig. 7F, oligomycin has a stronger effect on fast FIB). The authors should explain the discrepancy and what it really means.

The differential effects of antimycin vs. oligomycin on fast vs. slow fibroblasts are indeed an interesting finding from our data. Although antimycin and oligomycin are both respiratory chain inhibitors, their target complexes have very different functions. Antimycin is an inhibitor of complex III of the respiratory chain, which required for the flow of electrons to complex IV, thus oxidation of substrates and establishment of membrane potential. Antimycin therefore blocks the setting up of the proton gradient at the mitochondrial inner membrane and in turn blocks the entire respiratory chain including ATP-production. Oligomycin on the other hand specifically blocks the proton channel of the ATP-synthase, which lies at the end of the respiratory chain in complex V. Complex V has the function to not only reduce membrane potential, which allows the continuous function of the electron transport chain, but also to produce ATP. Therefore, the difference in response of fast vs. slow fibroblasts to complex V inhibition by oligomycin could be explained by cells relying differently on OXPHOS for ATP production.

Overall, it is therefore not inconceivable that the two drugs can have differential effects on the diverse and mechanistically distinct biological mitochondrial functions such as proliferation, cell survival and cellular state as indicated by expression of the mesenchymal marker N-Cadherin. We have added explanatory sentences to the results section on this topic. Nevertheless, we believe that it would go beyond the scope of this study to further elucidate the detailed reason for this difference in drug-response we observed.

8. Figure 7C, the label “Drug effect log2(DMSO/drug)” is ambiguous. What is being measured should be clearly stated in the figure or caption. Figure 7E is dim and hard to see. The cadherin staining should be quantified.

We have added the detailed explanation of measurement in the caption of Figure 7C. Furthermore, we have enhanced the contrast in order to make Figure 7E more visible and have provided a quantification of the N-Cadherin staining intensity, which confirmed our conclusion that oligomycin has a specific effect on fast fibroblasts regarding N-cadherin regulation.

9. Figure S6B, for the same protein, experiments group and the control (e.g. ESC as the positive control for cadherin) should be on the same gel.
As requested, we have replaced the Western Blot with the original Western image, which shows that all samples were loaded on the same gel and additionally shows results for double-drug treatment, which we left out originally for simplicity reasons. Double drug treatment mirrored the effect of antimycin treatment alone by not strongly affecting N-cadherin protein levels in fast fibroblasts. This can be explained by antimycin treatment (complex III) acting upstream of oligomycin treatment (complex V) in the electron transport chain and therefore being the dominant drug in the double-treatment.
Other minor issues:
I. In main text, abbreviation “FACS” first appeared at Line 119 but the abbreviation was introduced at Line 160. In Figure 1(E, F) caption, abbreviation “BrdU” was used but introduced later in Materials AND Methods (Line 586).
II. The metric prefix “micro” was written as “u” instead of “μ” at Line 560 and 603, which is not a SI recommendation.
III. It’s recommended to leave a space between a number and its unit, e.g., at Line 560, 572, 586, 603.
In Line 592, there should be a space between “0.5%” and “Triton”.
IV. Line 679, “viability” was marked bold for no obvious reason.
V. Line 442 and 444, Figure 7E and S6A-C are referenced. However, Figure S6 has no panel C.
VI. Figure 4A, the gene name “KI67” in the legend is inconsistent with the “Ki67” in the caption and main text.

We thank the reviewer for careful reading and pointing out these errors, which have now all been fixed.

**Reviewer #3**: This is a well-written manuscript with really fantastic results.

There is strong experimental and computational work, so it will be quite well-received by PLoS Computational Biology readers. Indeed, the results the authors have shown “a nearly universal signature that is strongly associated with proliferation” will be of interest to a broad reader base spanning across biological and computational fields.

I also appreciated that, in applying their approach across organisms/data, the authors demonstrated the “overfitting” used by other methods of proliferation estimation that were based on single-species data. This level of cross-species validation is truly remarkable.

That said, there were a couple of points that would have greatly increased my comprehension of the results from an initial read that I recommend authors and editors consider. (In addition there are a few minor points/typos.)

We thank the reviewer for the enthusiastic reception of our manuscript and the constructive suggestions in order to improve it.

1) I found it a bit disappointing that, in my view, that details of the critical achievement of the work: the establishment of a proliferation score, was relegated to the methods section.

I’m a mathematical modeler, so at least from my point of view the proliferation score itself is a result of the paper and merits greater discussion in the main text.

Although it relies on ssGSEA, an established method, some details within the manuscript of how the score itself is calculated would have been appreciated.

Thanks for the suggestion, we now explain the calculation of proliferation score in the main text (“We then applied ssGSEA(64), a rank….”).

2) To that end, reading the methods it appears that the authors consider only the genes with a correlation of 1 to proliferation in calculating their score. While clearly, this results in a highly predictive and not-overfit model, this criteria seemed quite strict.

Could the authors elaborate if, perhaps within a species, there are genes that are close to perfect correlation that would increase their predictive power? Or once you move outside perfect correlation do you encounter overfitting?

Yes, this is the case and reviewer 2 asked a similar question (specific comment 3). We do use both positively and negatively correlated genes to predict yeast growth rate in another study (unpublished), where we get better results than when using only positively or negatively corelated genes. But in this study, we first find that ribosome-biogenesis gene sets and proteasome related gene sets enriched in fast proliferating cells among fibroblasts, ESCs and yeast. None of the gene sets were enriched in slow proliferating cells for all fibroblasts, ESCs and yeast **(Figure 3C, D)**. This suggests that most of those negatively correlated genes are cell type specific or not stable, and thus might not be good candidates for proliferation prediction across cell types or species.

In general, to avoid overfitting, which had been a major problem in previous papers, we tried to avoid any arbitrary values (thresholds for correlation, deviation from default parameters in software, custom gene-sets, etc).

3) I am not an experimentalist, but have worked with CFSE data as a modeler. So I had some questions about these experiments.

Were the cells at the start were synchronized in some way? It seemed to me that heterogeneity in the cell cycle at the start of the experiment would lead to complications in identifying the “fast” from “slow” cells as you might capture who was “lucky” enough to be at the right point in the cell cycle.

The cells were not synchronized, but a G0/G1 subpopulation was obtained by sorting via Hoechst staining, and a homogenously stained population was selected by sorting 20% of cells on the peak of the CFSE signal and replating at the start of the experiment.

Again, I am familiar with mathematical approaches to estimating proliferation rates from CFSE data (such as described in <https://www.ncbi.nlm.nih.gov/pmc/articles/PMC3196292/>). The CFSE data in this manuscript (figure 1) is far “cleaner” than I am used to seeing from these previously published studies. If there non-standard experimental procedures which produced this cleaned data, I would recommend they be highlighted in the main text.

We thank the reviewer. We generated a homogenously stained starting population by FACS sorting on the peak of the CFSE signal. The study mentioned by the reviewer did not do that, but rather used non-sorted CFSE-stained cells, which show a more heterogenous pattern due to unequal CFSE uptake during the staining procedure.

We mention this in the results “the initial CFSE signal is independent of initial cell-to-cell variation in dye uptake or protein content, as the initial distribution is determined by the FACS gate.”

Minor Points:

• Figure 2: Panels (B) and (C), the caption should define what the “grey” regions around the linear fit. (Is this a 90% confidence band? 95% confidence band?)

Yes, is it 95% confidence band, and we have added it to the figure legend.

• Line 109: Is the first time in the main text that ESCs are used, I would recommend spelling this out.

Done.

• Line 139: “cause” should be “causes”

Done.

• Line 732: I was I was confused by what was meant by “proliferation rate” in the correlation? Is this just the normally calculated doubling time of a sample?

Yes, it is doubling time.

• Lines 793: The sentence beginning “We just sum up…” is quite long and hard to parse. I recommend putting a period after “proliferation signature score” and making a new sentence beginning “We do not use ssGSEA…”

Done.

**Have the authors made all data and (if applicable) computational code underlying the findings in their manuscript fully available?**
The [PLOS Data policy](https://journals.plos.org/ploscompbiol/s/materials-and-software-sharing) requires authors to make all data and code underlying the findings described in their manuscript fully available without restriction, with rare exception (please refer to the Data Availability Statement in the manuscript PDF file). The data and code should be provided as part of the manuscript or its supporting information, or deposited to a public repository. For example, in addition to summary statistics, the data points behind means, medians and variance measures should be available. If there are restrictions on publicly sharing data or code —e.g. participant privacy or use of data from a third party—those must be specified.

Reviewer #1: Yes

Reviewer #2: Yes

Reviewer #3: Yes

PLOS authors have the option to publish the peer review history of their article ([what does this mean?](https://journals.plos.org/ploscompbiol/s/editorial-and-peer-review-process#loc-peer-review-history)). If published, this will include your full peer review and any attached files.


If you choose “no”, your identity will remain anonymous but your review may still be made public.


**Do you want your identity to be public for this peer review?** For information about this choice, including consent withdrawal, please see our [Privacy Policy](https://www.plos.org/privacy-policy).

Reviewer #1: No

Reviewer #2: No

Reviewer #3: No

Figure Files:

While revising your submission, please upload your figure files to the Preflight Analysis and Conversion Engine (PACE) digital diagnostic tool, [https://pacev2.apexcovantage.com](https://pacev2.apexcovantage.com/). PACE helps ensure that figures meet PLOS requirements. To use PACE, you must first register as a user. Then, login and navigate to the UPLOAD tab, where you will find detailed instructions on how to use the tool. If you encounter any issues or have any questions when using PACE, please email us at [figures@plos.org](mailto:figures@plos.org).

Data Requirements:

Please note that, as a condition of publication, PLOS' data policy requires that you make available all data used to draw the conclusions outlined in your manuscript. Data must be deposited in an appropriate repository, included within the body of the manuscript, or uploaded as supporting information. This includes all numerical values that were used to generate graphs, histograms etc.. For an example in PLOS Biology see here: <http://www.plosbiology.org/article/info%3Adoi%2F10.1371%2Fjournal.pbio.1001908#s5>.

Reproducibility:

To enhance the reproducibility of your results, we recommend that you deposit your laboratory protocols in [protocols.io](http://protocols.io/), where a protocol can be assigned its own identifier (DOI) such that it can be cited independently in the future. Additionally, PLOS ONE offers an option to publish peer-reviewed clinical study protocols. Read more information on sharing protocols at <https://plos.org/protocols?utm_medium=editorial-email&utm_source=authorletters&utm_campaign=protocols>

References:

Review your reference list to ensure that it is complete and correct. If you have cited papers that have been retracted, please include the rationale for doing so in the manuscript text, or remove these references and replace them with relevant current references. Any changes to the reference list should be mentioned in the rebuttal letter that accompanies your revised manuscript.

*If you need to cite a retracted article, indicate the article’s retracted status in the References list and also include a citation and full reference for the retraction notice.*
